# Effects of Loaded End Distance and Moisture Content on the Behavior of Bolted Connections in Squared and Round Timber Subjected to Tension Parallel to the Grain

**DOI:** 10.3390/ma13235525

**Published:** 2020-12-03

**Authors:** Antonin Lokaj, Pavel Dobes, Oldrich Sucharda

**Affiliations:** 1 Department of Structures, Faculty of Civil Engineering, VSB-Technical University of Ostrava, 70800 Ostrava-Poruba, Czech Republic; antonin.lokaj@vsb.cz; 2 Department of Building Materials and Diagnostics of Structures, Faculty of Civil Engineering, VSB-Technical University of Ostrava, 70800 Ostrava-Poruba, Czech Republic; oldrich.sucharda@vsb.cz; 3 Centre of Building Experiments, Faculty of Civil Engineering, VSB-Technical University of Ostrava, 70800 Ostrava-Poruba, Czech Republic

**Keywords:** connection, timber, test, bolt, steel plate, moisture content, failure

## Abstract

This article presents the results of static tests on bolted connections in squared and round timber with inserted steel plates. The experiment evaluates structural timber connections with different distances between the fastener and the loaded end at different moisture contents. Specimens were loaded by tension parallel to the grain and load–deformation diagrams were recorded. Fifty-six specimens with three different distances between the fastener and the loaded end, at different moisture contents, were tested. The results were statistically evaluated using regression analysis, complemented with load–deformation curves, and compared with calculations according to the valid standard for design of timber structures. A decrease in the evaluated load-carrying capacity with increasing moisture content was confirmed experimentally. A slight increase in the evaluated load-carrying capacity with increasing fastener distance from the loaded end was found.

## 1. Introduction

Currently, the use of timber as a building material is becoming increasingly popular. The general trend of using natural, renewable and easily recyclable materials in construction practice contributes to this fact. Increasingly, environmental requirements and long-term sustainability in construction are gaining prominence and application (e.g., in buildings [1], bridges and footbridges [2]). Timber structures, which are commonly used as a substitute for steel and concrete structures, considerably mitigate the impact on the environment thanks to their smaller carbon footprint [3].

**Figure 1:**
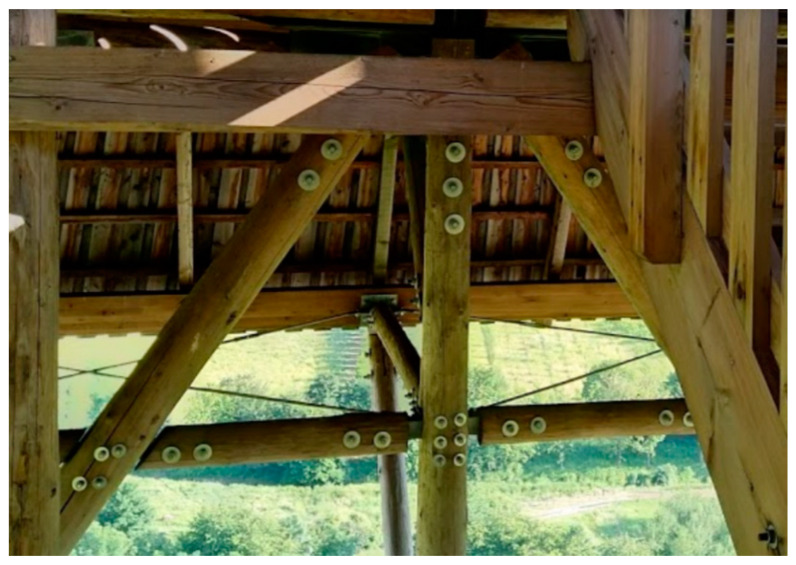
Practical use of connections in timber structures with mechanical fasteners and steel plates.

One of the most important areas in the design of timber structures is connections [4,5,6]. Connections affect the overall composition of the load-carrying structure and dimensions of the main load-carrying members [7]. The load-carrying capacity and the stiffness of connections are crucial to the serviceability and durability of the whole structure, especially for large-span structures with many connections [1,7] and structures with heavily loaded connections [2]. The most common types of connections in timber structures use metal mechanical fasteners, which are often used in combination with steel plates slotted into cut-outs in timber members (see Figure 1) [8,9,10,11,12,13,14,15,16].

The stiffness of connections is closely related to the physical and mechanical properties of timber and fasteners [17,18], as well as to the design of individual details (i.e., placement of individual fasteners and the whole connection geometry) [19,20,21]. The influence of connection stiffness manifests in increased deformations and redistribution of internal forces, which can significantly affect the static design of the whole load-carrying structure [7].

One of the most important physical property of timber that significantly affects its strength and deformation properties [13,22], and its durability especially [23], is the moisture content. Due to atmospheric influences, timber, as a hygroscopic material, constantly exchanges moisture with the surrounding environment [24,25]. The effects of moisture content on the load-carrying capacity and stiffness of connections has been proven in long-term research by forest researchers in the US [26]. Fluctuations in moisture content also result in volume changes (shrinkage and swelling), which, due to the formation of cracks and reduction in embedment strength, negatively affects the load-carrying capacity of connections [25]. As a result, it is necessary to consider the influence of the environmental conditions (especially humidity) under which the structure will be built, as well as the type of load that will be carried. The hygroscopic properties of timber can be improved, for example, by temperature treatment [24].

The contemporary European standard for the design of timber structures EN 1995-1 (Eurocode 5) [27] includes calculations for timber-to-timber or steel-to-timber connections using mechanical fasteners such as bolts, dowels, nails, screws, etc. Timber-to-timber connections also include combinations of different timber materials [28]. Calculations of load-carrying capacity and connection stiffness do not take into account the edge and end distances nor spacings for fasteners; they are only given as the minimum standard requirement to prevent brittle failure [19,29,30]. Rahim et al. investigated the influence of double-shear steel-to-timber connection geometry (different spacings, end distances) on the initial stiffness [20]. It was found that the initial stiffness can be affected by many factors that are not taken into account in Eurocode 5. Awaludin and Saputro pointed to the possibility of using values smaller than those recommended for the distance between the fastener and the loaded end for a dowel-type connection in laminated veneer lumber [21].

Eurocode 5 and current design codes in many other countries are not optimal, although they are conservative [31]. The European standard only describes three modes, which do not represent total connection failure. Those failure modes are based on the so-called European yield model according to Johansen’s theory from 1949 [32], which represents ductile failure modes. The main objective under this model is to design connections for which ductile failure comes before brittle failure, i.e. to design connections with the highest possible deformation capacity to prevent an unexpected collapse [33]. This approach is supported by several scientific studies [19,29,34]. Various connection reinforcement methods have also been used to prevent brittle splitting [12,14,35].

Current research indicates a disagreement between experiments (brittle failure) and design standards (ductile failure) [30,31]. Real total failure of bolted connections occurs due to tension or shear stress [36] and in some cases (multiple shear connections with several slotted-in steel plates) may even be crucial [16]. Brittle failure modes of steel-to-timber bolted connections loaded by tension parallel to the grain were described in [34]. Jorissen developed a new design approach based on fracture mechanics [29].

In this study, static tests were performed on bolted connections in squared and round timber specimens with inserted steel plates. Several connection variants with different distances between the fastener and the loaded end and with different moisture contents (square timber only) were chosen in order to verify if the real load-carrying capacity was influenced by distance. The specimens were subjected to tension parallel to the grain, until the connections failed.

## 2. Research Significance

Determining the load-carrying capacity of connections in timber structures and applying new knowledge to the European standard for timber structures design [27] are areas which are still under development. This research describes and compares the behavior of connections in squared and round timber, loaded in tension parallel to the grain, with different distances between the fastener and the loaded end and for different moisture contents.

Some studies from the scientific literature have investigated the design and behavior of dowel-type connections in timber structures, mainly based on experimental research. For example, important findings can be found in publications [4,5,6]. The relevance of this issue is also evidenced by the number of research papers written by authors mentioned in this paper.

The aim of this study is also to build on previous research activities conducted at the Faculty of Civil Engineering of the VSB Technical University of Ostrava by Klajmonova et al. A series of experiments was carried out to compare the load-carrying capacity of bolted connections with inserted steel plates, both for squared timber and round timber, but always for the same placement and number of fasteners [8,9,10,11]. Methods of effective reinforcement for bolted connections subjected to brittle failure were also designed and tested [12].

Some important new findings from our experimental testing have already been published and discussed at international conferences [37,38] and should be further extended.

Finally, the influence of moisture content on the behavior of a similar type of connection (bolt M12, glued laminated timber) loaded parallel to the grain has recently been investigated [13].

## 3. Design and Analysis of Timber Structure Connections in Europe

In most European countries, according to the current legislation, timber structures are designed and constructed based on Eurocode 5 [27]. Selected important knowledge about connections in timber structures is described in this chapter (for more detailed information, see the EN 1995-1 standard).

The geometric arrangement of fasteners in a connection (spacings, edge and end distances) has to meet minimum values to achieve the expected strength and stiffness. The most important value is the end distance from the loaded end (designation *a*_3,t_ according to the standard). The minimum value depends on the fastener diameter *d* and is given according to Equation (1).

(1) $$a_{3,t}=\max\left(7d;80\;mm\right)$$

When determining the characteristic load-carrying capacity of connections with dowel-type fasteners made of metal, it is necessary to take into account the embedment strength of the timber, the yield strength of the fastener and less commonly the withdrawal strength of the fastener. 

Connections are distinguished based on the type of materials to be connected. There are timber-to-timber, panel-to-timber and steel-to-timber connections.

**Figure 2:**
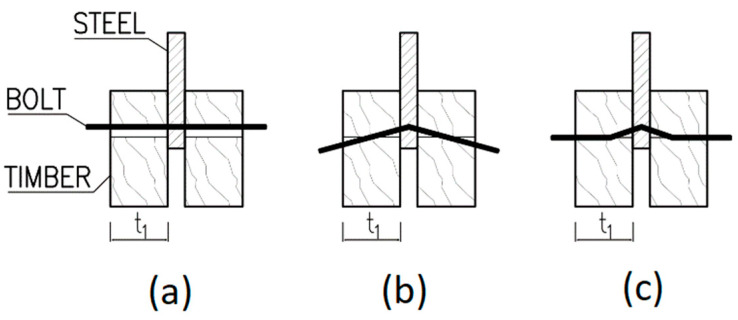
Failure modes for steel-to-timber connections: (**a**) Embedment of the timber; (**b**) Bending of the fastener; (**c**) Combination of both failures.

For a double shear steel-to-timber connection with a steel plate of any thickness as the central member, the characteristic load-carrying capacity of one fastener for a single shear plane is determined as the minimum value according to Equation (2) for the failure modes shown in Figure 2 (failure mode *a* represents embedment of the timber, failure mode *b* represents bending of the fastener and failure mode *c* is the combination of both mentioned failure modes) or in [27]. The first member in the formula is the load-carrying capacity based on the Johansen yield theory [32] and the second member represents the so-called rope effect [39,40], which in Eurocode 5 has to be limited to 25% of the Johansen part for bolts. If the contribution from the rope effect is unknown, then it is taken as zero.

(2) $$F_{v,Rk}=\{\begin{array}{cc} f_{h,1,k}\cdot t_{1}\cdot d & \left(a\right)\\ f_{h,1,k}\cdot t_{1}\cdot d\cdot \left[\sqrt{2+\frac{4\cdot M_{y,Rk}}{f_{h,1,k}\cdot d\cdot t_{1}^{2}}}-1\right]+\frac{F_{ax,Rk}}{4} & \left(b\right)\\ 2.3\cdot \sqrt{M_{y,Rk}\cdot f_{h,1,k}\cdot d}+\frac{F_{ax,Rk}}{4} & \left(c\right) \end{array}$$

here,

*F_v,Rk_* is the characteristic load-carrying capacity for a single shear per fastener [N];

*f_h,k_* is the characteristic embedment strength in the timber member [N/mm^2^];

*t*_1_ is the smaller of the thicknesses of the timber side member [mm];

*d* is the diameter of the fastener [mm];

*M_y,Rk_* is the characteristic yield moment of the fastener [N/mm];

*F_ax,Rk_* is the characteristic withdrawal capacity of the fastener [N].

For bolts, the characteristic value of the yield moment depends on the bolt diameter *d* and the characteristic tensile strength *f_u,k_* and is determined according to Equation (3).

(3) $$M_{y,Rk}=0.3\cdot f_{u,k}\cdot d^{2.6}$$

For bolts, the characteristic embedment strength value in timber parallel to the grain depends on the bolt diameter *d* and the characteristic density of timber *ρ_k_* and should be calculated according to Equation (4).

(4) $$f_{h,0,k}=0.082\cdot \left(1-0.01\cdot d\right)\cdot \rho_{k}$$

Moisture content is given by the ratio of water weight to dry weight. When stored in air, European spruce timber dries to the so-called hygroscopic equilibrium moisture content (around 20%). The ideal moisture content for spruce timber used in exterior construction is considered to be 12% [2].

The effect of moisture on the mechanical properties of timber is significant. As the moisture content increases, the strength and stiffness of the timber decrease. The linear relationship between mechanical properties and moisture content can be considered to be in the range of 8% to 20% for moisture content [26].

When determining design values of mechanical properties according to [27], the influence of air humidity can be taken into account by assigning the structure to one of the three service classes, which affects the choice of the relevant modification factor *k_mod_* and deformation factor *k_def_*.

## 4. Methodology of Testing

### 4.1. Description of Materials for Test Specimens

**Figure 3:**
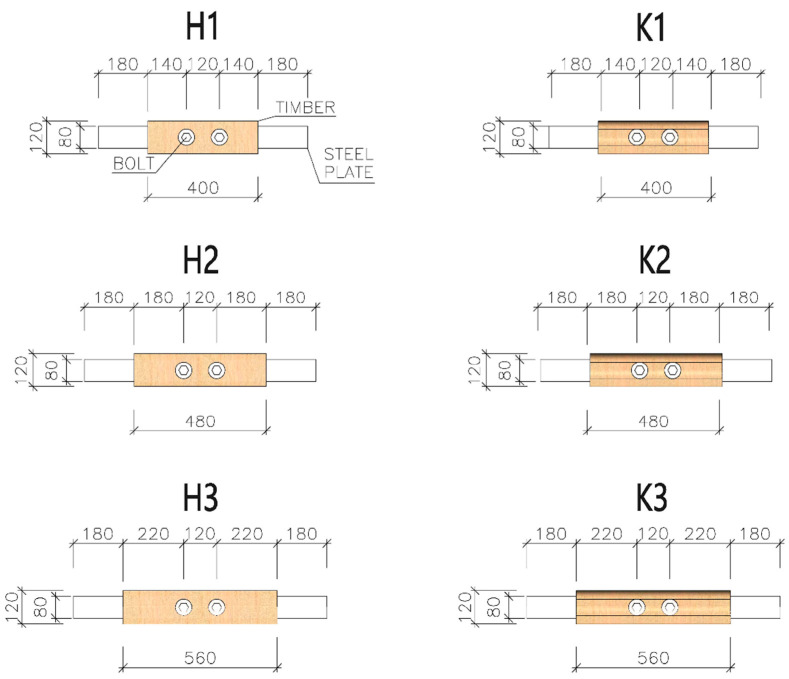
Types of specimens for tension tests parallel to the grain.

**Figure 4:**
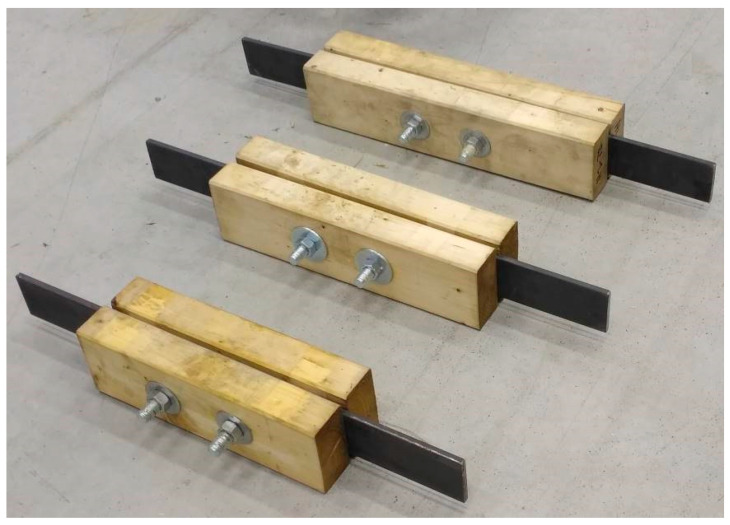
All types of squared specimens before testing.

For tension tests parallel to the grain, specimens were made of squared timber with cross-sectional dimensions of 60 × 120 mm and round timber with a diameter of 60 mm. All specimens consisted of two equal parts due to easier production of final specimens (placement of steel plates between individual parts). The specimens were made of spruce timber of the C24 strength class (determined on the basis of the standard [41]). Specimens of three different lengths were tested (see Figure 3 and Figure 4): 400 mm (designation H1 and K1), 480 mm (designation H2 and K2) and 560 mm (designation H3 and K3). The circular holes for placing fasteners had a diameter of 20 mm. 

High tensile bolts, grade 8.8 (*f_y_* = 640 MPa, *f_u_* = 800 MPa) with a diameter of 20 mm, were used as fasteners. The bolts were placed at three different distances from the loaded end: 140 mm (=7× bolt diameter), 180 mm (=9× bolt diameter) and 220 mm (=11× bolt diameter). The axial spacing between two bolts in one specimen was 120 mm.

**Figure 5:**
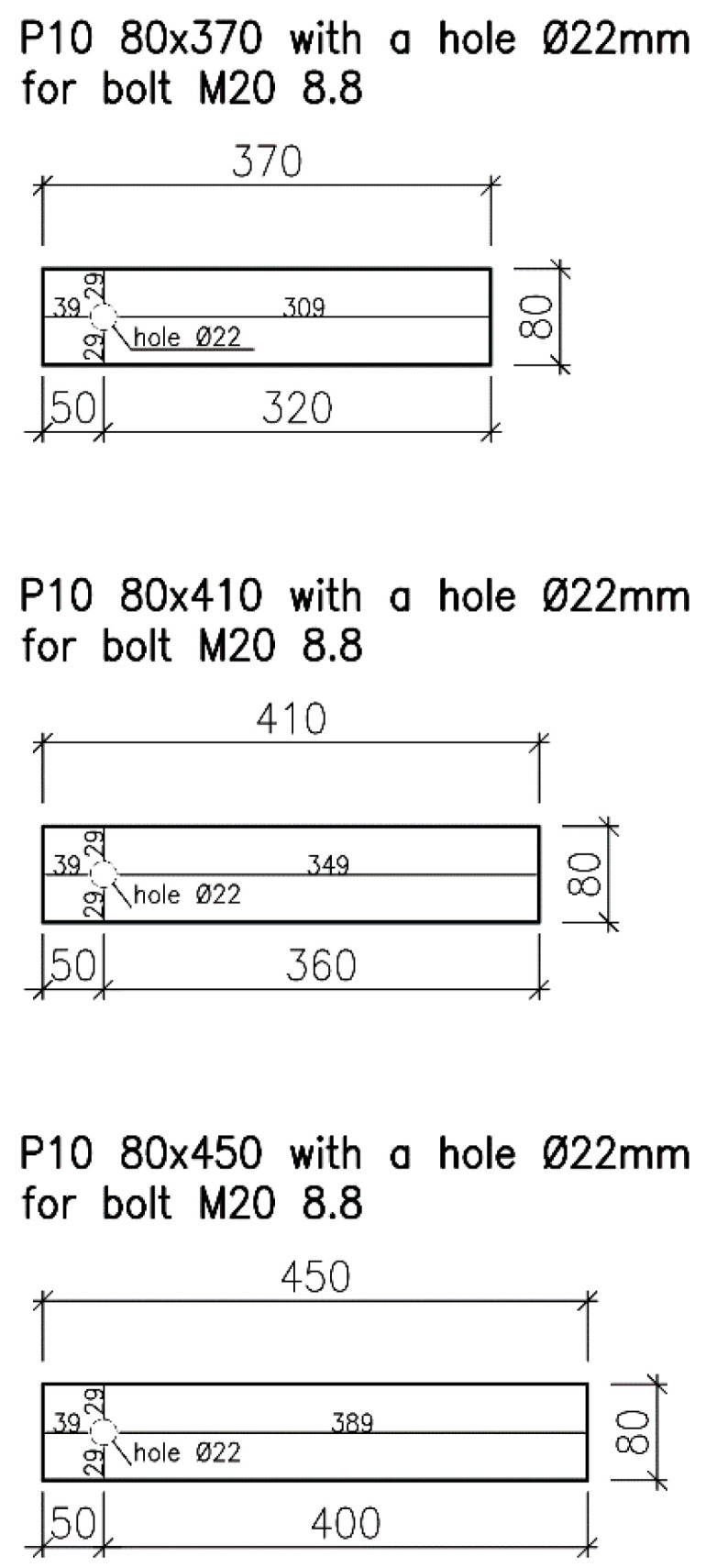
Dimensions of steel plates for tension tests parallel to the grain.

**Figure 6:**
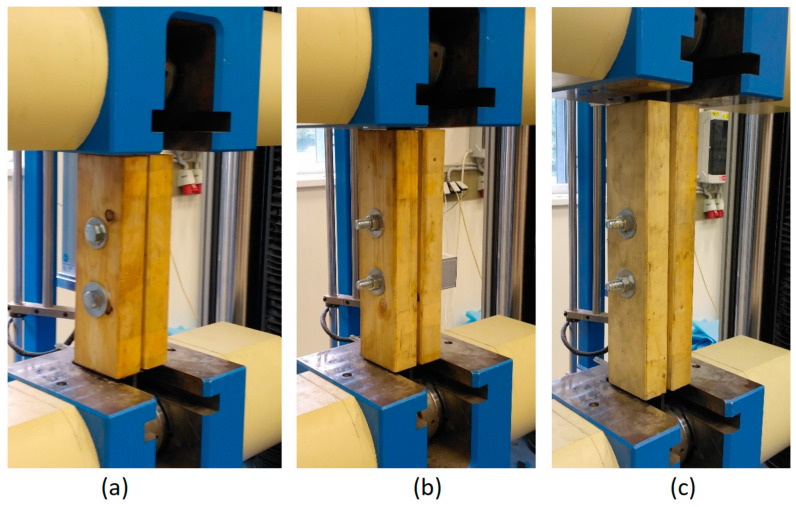
Specimens of squared timber clamped in the jaws of the testing machine: (**a**) H1 specimen–length 400 mm; (**b**) H2 specimen–length 480 mm; (**c**) H3 specimen–length 560 mm.

The steel plates (see Figure 5) were made of structural-grade steel S235J0; their thickness was 10 mm. The dimensions of the steel plates also varied depending on the distance between the fastener and the loaded end. The protruding part of the plates needed for clamping specimens into the jaws of the testing machine was 180 mm long (see Figure 6). The plates were provided with circular holes with a diameter of 22 mm for placing bolts (a clearance of 2 mm was left).

For detailed specifications of dimensions, see Figure 3 and Figure 5 and Table 1.

All timber specimens were produced and industrially manufactured at approximately equilibrium moisture content (about 12%). Some squared timber specimens were then moistened. The aim of this was not to achieve an accurate level of moisture in the tested timber, but to simulate adverse conditions that could occur during the service life of an actual supporting structure using these types of elements and connections. The specimens were exposed to changing atmospheric influences for six months (February–July) without sunlight. Some were protected against rainwater penetration inside (this corresponds to specimens with a moisture content between approximately 18% and 22%). Some were not protected (this corresponds to specimens with a moisture content of about 30% and higher).

### 4.2. Description of the Testing Course

Several non-destructive tests were carried out before the main test to determine the quality of timber material, its moisture content and its density. The test specimens were weighed on a laboratory scale and their dimensions and moisture content were measured. Based on these data, the bulk density of the specimens and the density derived therefrom were determined according to [42,43]. Moisture content was measured using a capacitive material moisture meter according to [44].

The experiments were performed using the LabTest 6.1200 electromechanical testing machine (LABORTECH s.r.o., Opava, Czech Republic) with 1200 kN maximum electrohydraulic cylinder force (see Figure 6). The machine and testing procedure were controlled by computer software.

The specimens were clamped into jaws with special serrations for better adhesion, so the slip in the jaws was eliminated. The lower jaw was static, the upper jaw was movable. The tested connections were subjected to an axial tensile load with possible additional effects from imperfections for which no significant effect on the evaluated parameters was expected. Possible imperfections included only geometric inaccuracies in cutting and drilling of the timber specimens. The arrangement and loading of the test specimens were designed to correspond to the actual state of connections in real supporting structures. The aim was not to simulate ideal conditions but to verify the behavior of the connections under realistic conditions. 

**Figure 7:**
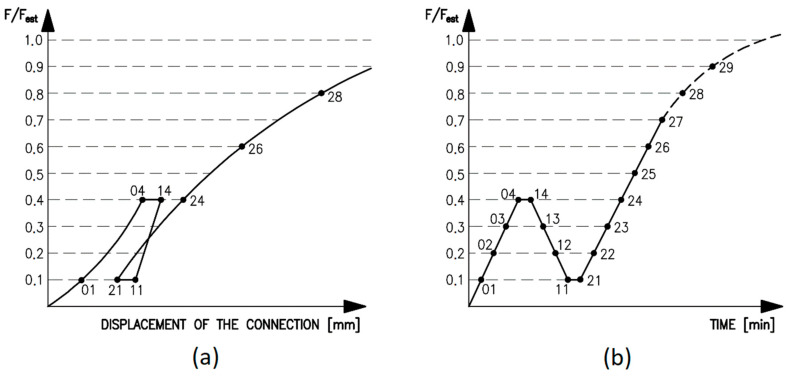
(**a**) Idealized load–displacement diagram and measured values; (**b**) The course of testing during time.

For this reason, the bolts were also tightened by hand, using a spanner without specific tightening force, to eliminate the rope effect [39]. The tensile load was generated by electrohydraulic cylinders with a capacity of 1200 kN. During the test, the time, tensile force and deformations of the connection in the longitudinal direction (i.e., cross-head displacement) were continuously recorded. The loading course (see Figure 7) was carried out in accordance with the standard for testing the connections in timber structures with mechanical fasteners [45]. The following loading procedure was prescribed:

1. estimate the maximum force *F_est_*, for the tested connection based on experience, calculation or pre-tests;
2. load the specimen to 40% of the estimated maximum force, 0.4·*F_est_*, then hold for 30 s;
3. decrease the load to 10% of the estimated maximum force, 0.1·*F_est_*, then hold for 30 s;
4. continue loading until the specimen fails.

**Figure 8:**
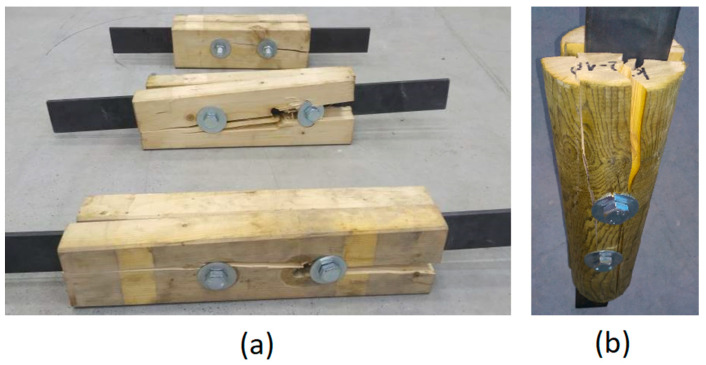
Specimens after failure: (**a**) Squared timber; (**b**) Round timber.

A constant loading speed was chosen (20 kN/min). The total testing time for one specimen was about 10 to 15 min until failure (see Figure 8).

### 4.3. Evaluation of the Testing

The measured displacements *ν*_01_, *ν*_04_, *ν*_14_, *ν*_11_, *ν*_21_, *ν*_24_, *ν*_26_, *ν*_28_ and the displacements at the maximum load were recorded. Based on the recorded data, it is possible to determine the initial displacement *ν_i_* = *ν*_04_, the modified initial displacement (Equation (5)), the permanent initial displacement (Equation (6)) derived therefrom and the initial slip modulus (Equation (7)) and the slip modulus (Equation (8)) derived therefrom [45].

(5) $$\nu_{i,mod}=\frac{4}{3}\cdot \left(\nu_{04}-\nu_{01}\right)$$

(6) $$\nu_{s}=\nu_{i}-\nu_{i,mod}$$

(7) $$k_{i}=0.4\cdot \frac{F_{est}}{\nu_{i}}$$

(8) $$k_{s}=0.4\cdot \frac{F_{est}}{\nu_{i,mod}}$$

## 5. Results

For this work, six types of bolted connections in squared timber and round timber with inserted steel plates were tested. Only squared timber specimens were tested with different levels of moisture content. In total, fifty-six specimens were tested.

**Figure 9:**
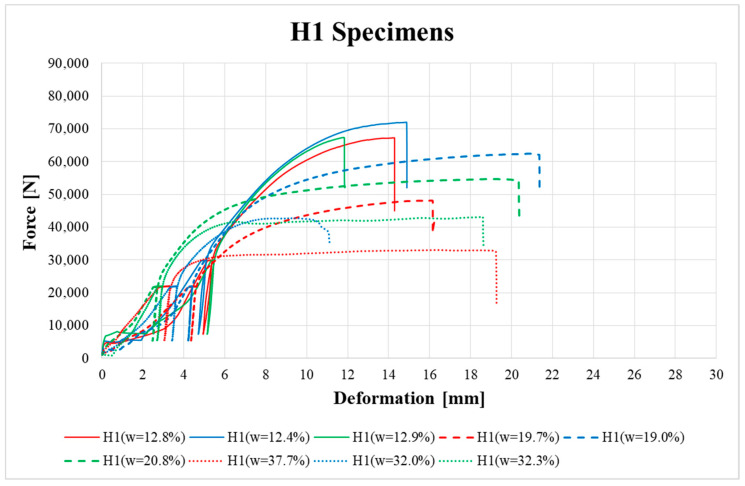
Load–deformation curves of H1 specimens.

**Figure 10:**
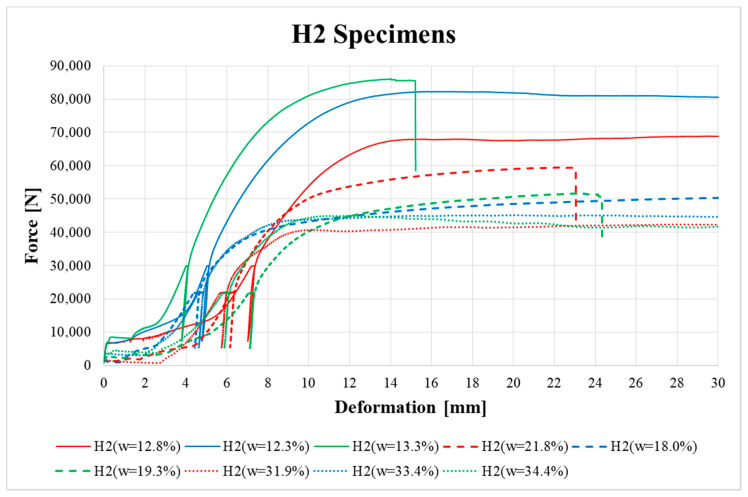
Load–deformation curves of H2 specimens.

**Figure 11:**
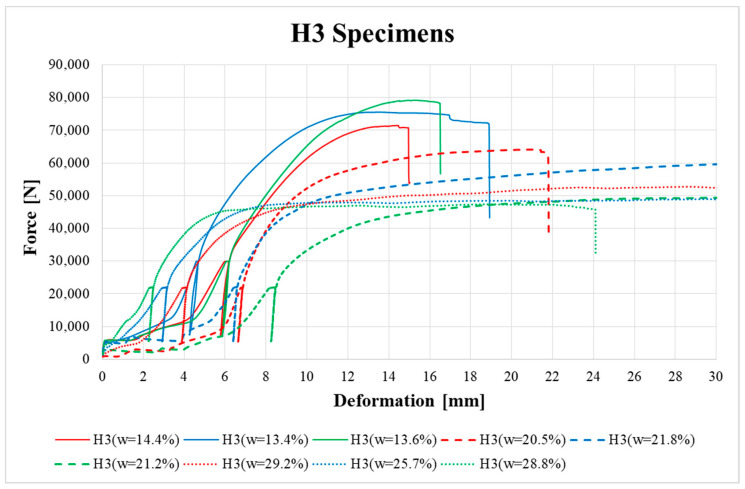
Load–deformation curves of H3 specimens.

**Figure 12:**
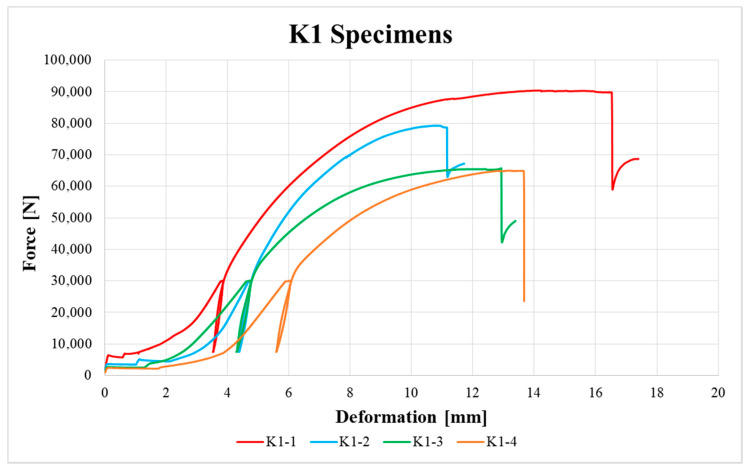
Load–deformation curves of K1 specimens.

**Figure 13:**
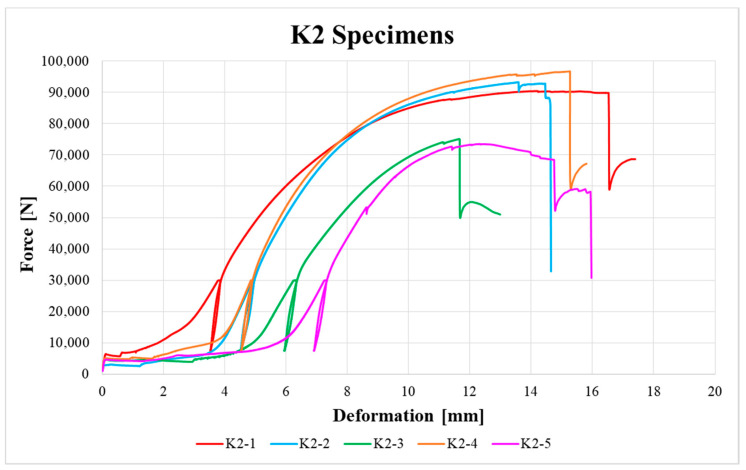
Load–deformation curves of K2 specimens.

**Figure 14:**
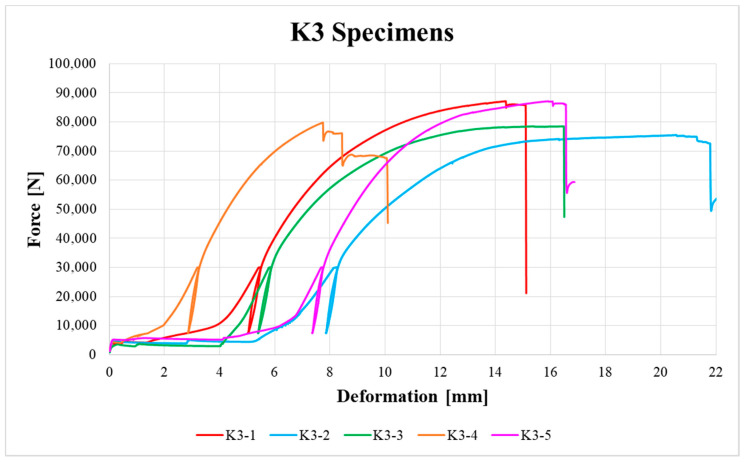
Load–deformation curves of K3 specimens.

Figure 9, Figure 10, Figure 11, Figure 12, Figure 13 and Figure 14 show the load–deformation curves of selected H1, H2, H3, K1, K2 and K3 specimens. In the graphs the initial loading at 40% of the estimated maximum force, the subsequent unloading to 10% of the estimated maximum force and the loading to failure can be seen. It is also possible to see the actual maximum force and the corresponding deformation at failure. The legends in the graphs for squared timber also contain information about the moisture content of the individual specimens.

**Figure 15:**
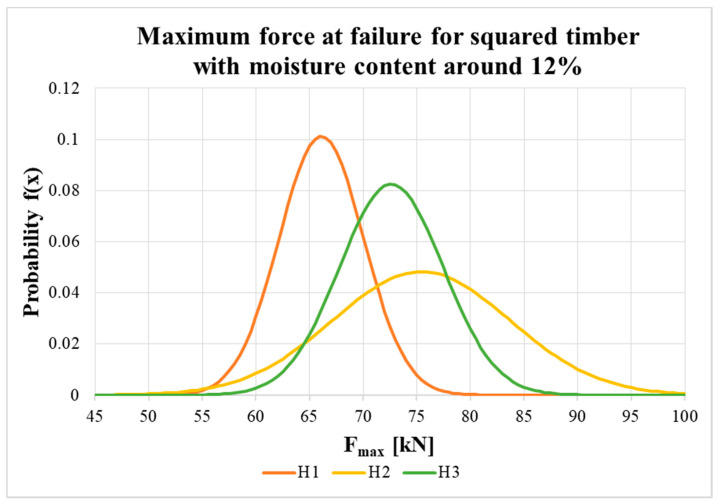
Probability density function of maximum force at failure for H1, H2 and H3.

**Figure 16:**
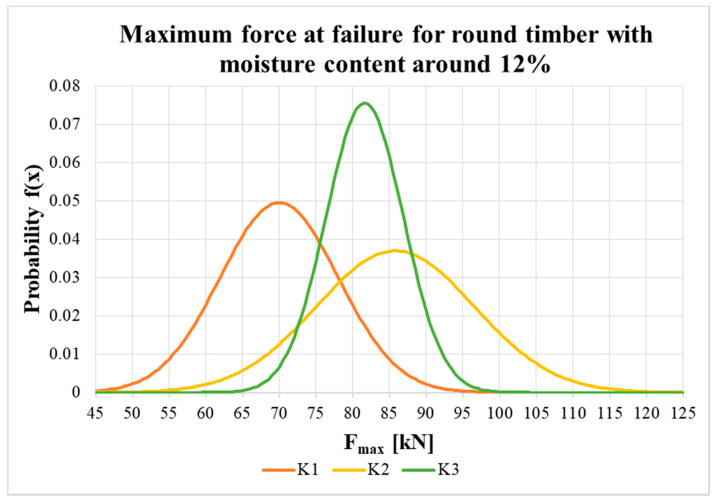
Probability density function of maximum force at failure for K1, K2 and K3.

Figure 15 and Figure 16 show the probability density function and the variance in load-carrying capacity of squared timber and round timber specimens with an equilibrium moisture content of about 12%. The statistical evaluation uses a normal distribution with input parameters, which were evaluated from the results of the experiment (see the average value and standard deviation in Table 2).

Table 2 shows statistically evaluated values of bulk density and moisture content for the timber material (the values given are for timber specimens during their manufacturing), the average maximum load-carrying capacity with standard deviation, the coefficient of variation and characteristic value based on testing, the calculated characteristic load-carrying capacity without contribution from the rope effect according to Eurocode 5 [27] (see the calculation below) and the values of the initial slip modulus and the slip moduli according to the above-mentioned formulas for all types (H1, H2, H3, K1, K2 and K3). The results were not normalized for the density data due to low variability in density. A normal distribution of test values was assumed in the statistical evaluation in accordance with [42,43].

here,

ρ is the bulk density of timber specimens; 

*w* is the moisture content of timber specimens;

*F*_max,test_ is the maximum force at failure based on test data (average value and standard deviation);

*F*_k,test_ is the characteristic force at failure based on test data (determined according to [42,43]);

*F*_k,EC5_ is the characteristic load-carrying capacity according to the standard [27];

*k*_i_ is the initial slip modulus of connections based on test data;

*k*_s_ is the slip modulus of connections based on test data.

In accordance with Eurocode 5 [27], a double shear steel-to-timber connection was considered (steel plate of any thickness as the central member). The characteristic value of the load-carrying capacity, without the contribution from the rope effect for the given type of connection, according to the above-mentioned standard is *F_v,Rk_ =* 46,528 N (twice 23,264 N). In order to calculate the load-carrying capacity, the necessary input values for C24 strength timber (determined on the basis of the standard [42]) and M20 grade 8.8 bolts were used. The calculation according to Eurocode 5 is given below in Table 3, where the failure mode *b* is decisive. 

To obtain the design values, it is necessary to recalculate the characteristic value using the modified factor for the duration of load and moisture content *k_mod_* (*k_mod_* = 1.1 for the 1st/2nd service class and *k_mod_* = 0.9 for the 3rd service class, both for instantaneous load duration) and the partial factor for the material properties *ɣ_M_* (*ɣ_M_* = 1.3 for connections).

**Figure 17:**
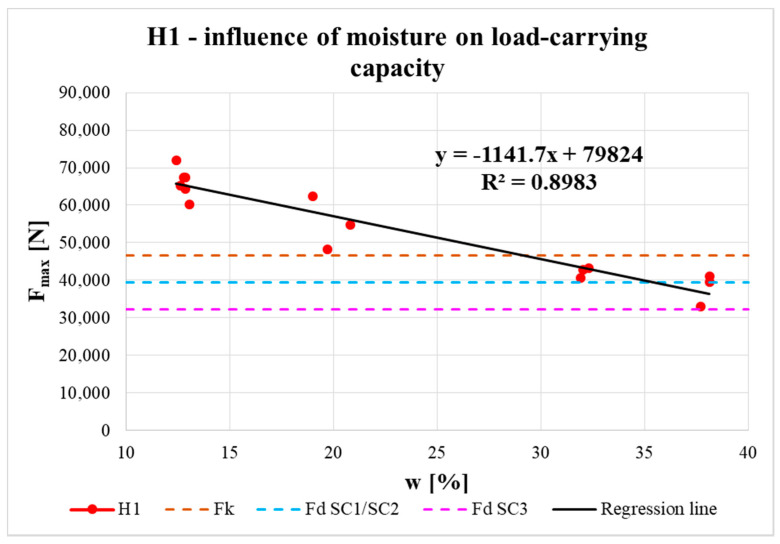
Influence of moisture content on load-carrying capacity of H1 specimens.

**Figure 18:**
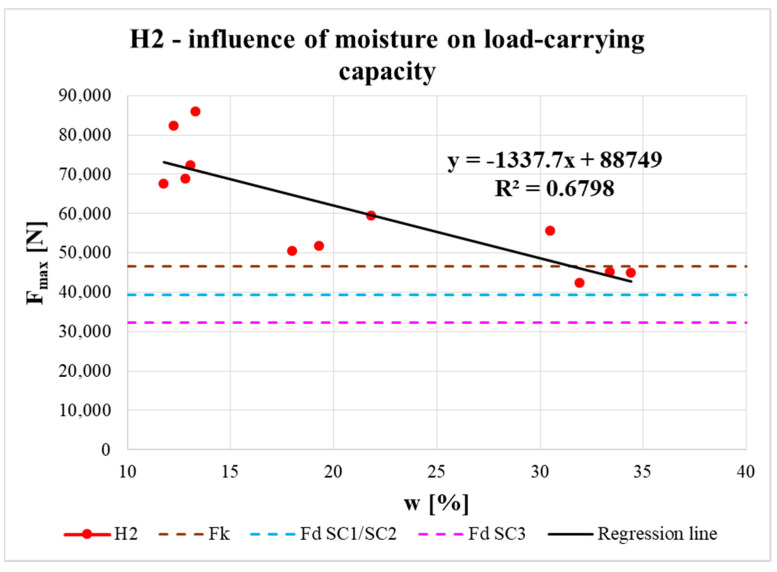
Influence of moisture content on load-carrying capacity of H2 specimens.

**Figure 19:**
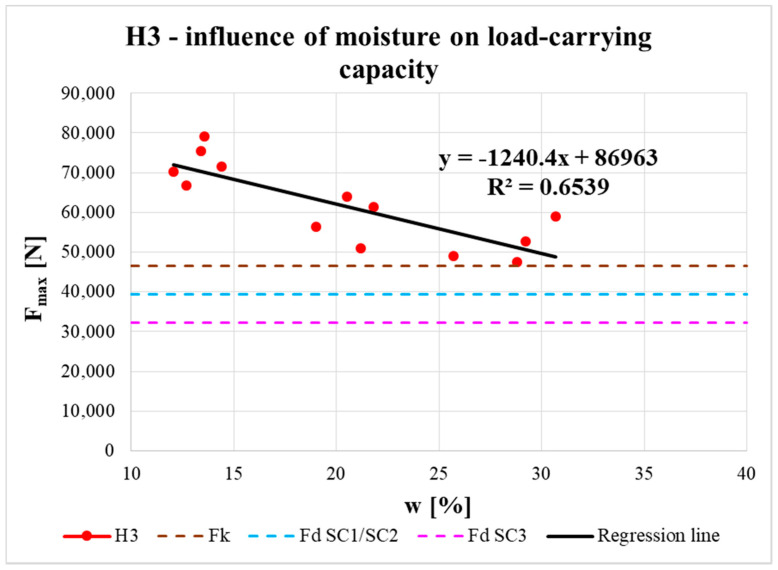
Influence of moisture content on load-carrying capacity of H3 specimens.

The effect of moisture content on the real load-carrying capacity of the connection is shown in Figure 17, Figure 18 and Figure 19. The obtained data are complemented with regression line equations and correlation coefficients. The graphs are also complemented with the characteristic load-carrying capacity *F_k_* and design load-carrying capacities for 1st/2nd service class *F_d_ SC1/SC2* (design values for both classes were the same) and 3rd service class *F_d_ SC3* for instantaneous load duration based on calculation according to the standard (see the calculation below).

**Figure 20:**
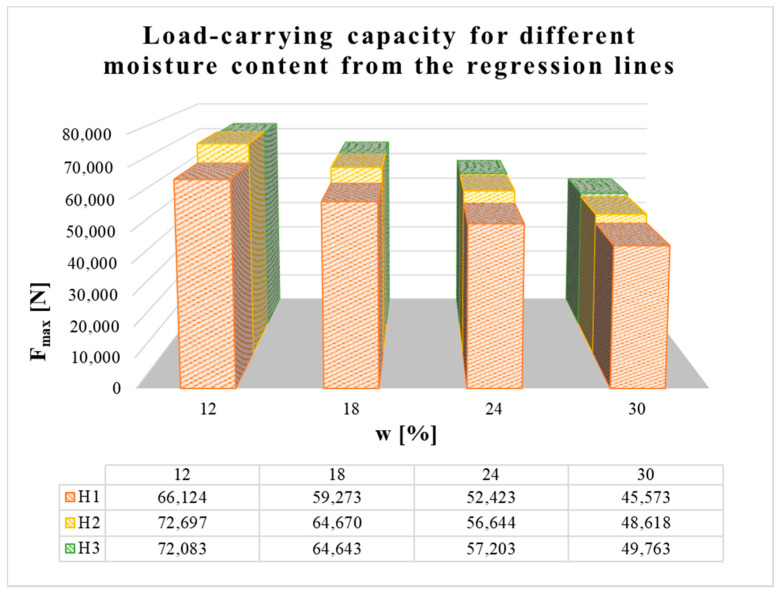
Load-carrying capacity for different moisture content from the regression lines.

Figure 20 shows the load-carrying capacities of all three types of connections in squared timber at different moisture content levels, which were obtained by putting those levels into the regression line from Figure 17, Figure 18 and Figure 19.

## 6. Discussion

From the initial loading, deformation of holes in timber members occurred until the specimens failed due to tension perpendicular to the grain. Failure always occurred by timber splitting under the bolt, when the tensile strength perpendicular to the grain was exceeded and cross-links between individual fibers in timber were broken (see Figure 8). This phenomenon can be attributed to the fact that timber under tension has little plasticity and is broken by brittle fractures. The failure in most specimens occurred after a visible embedment (ductile failure usually preceded brittle failure). The splitting crack spread very quickly in one half of a timber specimen (the other half remained only embedded) under one of the bolts and in some cases also between the bolts. Brittle failure indicated that higher capacities could be obtained and were not limited to the ductile failure modes from Eurocode 5 (depending on the embedment strength of the timber and yield moment of the fastener, see Section 3).

The maximum force values at specimen failure with moisture content around 12% (see Table 2) showed that, at the shortest distance between the fastener and loaded end (i.e., 7*d*), the average values were lowest for both the squared timber and round timber. For specimens with longer distances (i.e., 9*d* and 11*d*), the average load-carrying capacity was higher, and the highest value was surprisingly for middle-length specimens (H2 and K2 specimens). Higher load-carrying capacities were measured for round timber specimens than for squared specimens, probably due to the higher average bulk density of the timber (higher by between 4.1% and 11.8%). The results also show that there was no increase in real load-carrying capacity above the end distance 9*d*, so the use of a higher end distance has no practical significance.

However, in construction, useful values are so called characteristic values, evaluated as the 5% quantile of the approximated probability distribution of the measured values. Characteristic values are significantly influenced by the number of specimens (reduction in the number of test specimens, *k_s_(n)*, according to [43]) and variability in the measured values (sample standard deviation). The characteristic load-carrying capacities for all types of specimens were higher than the standard value for the double shear steel-to-timber connection (steel plate of any thickness as the central member). The highest values were found for H3 and K3 specimens (the longest distance of the bolt from the loaded end); for H3 specimens it was higher by 30.5% and for K3 specimens it was higher by 47.5%. Nevertheless, it is not possible to conclude there was a significant influence exerted by the distance between the fastener and the loaded end on the characteristic load-carrying capacity of the connection. This is complicated by the small number of tested specimens, which considerably affects the statistical evaluation of specimens with higher variance (especially specimens with coefficients of variation higher than 10%).

In addition, considerable variability can be seen in the slip moduli (see Table 2). The slip modulus *k_s_* was higher for round timber than for squared timber (almost twice as high), which is probably, again, due to the higher bulk density of round timber.

It is known that with increasing moisture content in timber elements (related to service class assignment), the load-carrying capacity of elements, and their connections, is reduced by the modification factor *k_mod_*. This decrease in real load-carrying capacity with increasing moisture content was also confirmed experimentally. The measured data could be approximated by a straight line using regression analysis (see Figure 17, Figure 18 and Figure 19). Correlation coefficients were evaluated (R = −0.95 for H1 specimens, R = −0.82 for H2 specimens, R = −0.81 for H3 specimens) and they indicated significant correlation between the examined parameters. The slopes of all the regression lines were quite similar, which indicates a similar effect of moisture content on load-carrying capacity for all types of specimens. The small differences were mainly due to the end distances.

Regression lines were then used to determine the load-carrying capacities for different moisture contents. The results show that at the shortest distance between the fastener and loaded end (i.e., 7*d*) the load-carrying capacity was lowest. For specimens with longer distances (i.e., 9*d* and 11*d*), the average load-carrying capacities were higher and the values for both distances were almost comparable. It can therefore be observed that with increasing distance between the fastener and the loaded end, there was a slight increase in the real load-carrying capacity (from the minimum standard value of 7*d* to the value of 9*d*). As the distance increased further, the increase in load-carrying capacity was practically negligible (from 9*d* to 11*d*). 

To assign the structure to service class 1, the moisture content of the built-in timber elements needs to be around the equilibrium moisture content of 12% (only for interiors). To assign the structure to service class 2, this moisture content must not exceed 20%. In cases where the moisture content is more than 20%, it is necessary to assign the structure to service class 3 [27]. Figure 17, Figure 18 and Figure 19 show that for all types of specimens with moisture contents around 12% (corresponding to service class 1) and 20% (corresponding to service class 2), the real load-carrying capacities were higher than the design value for service classes 1 or 2, even higher than the characteristic value according to the standard [27]. Furthermore, for specimens with the highest moisture content (corresponding to service class 3), the real load-carrying capacities were higher than the design value for service class 3 according to the standard [27]. For H2 specimens, the real load-carrying capacities were higher than the design value for service classes 1 or 2 and for H3 specimens they were even higher than the characteristic value. The values of real load-carrying capacities for H1 specimens with the highest moisture content point to the importance of carefully designing timber structures, especially exterior details (connections), because of the significant influence from atmospheric conditions, which can lead to rainwater flowing into improperly designed details and, subsequently, significantly decrease the real load-capacity capacity to the limit according to the standard [27].

With increasing moisture content in the squared timber specimens, a slight increase in the slip modulus can be observed, which appears to be due to the swelling of timber when it is saturated with water. However, no significant correlation was found between the slip modulus and moisture content dependence.

According to Figure 10 and Figure 11, specimens H2 and H3 also showed higher ductility in the final phase of loading before total brittle failure compared to specimen H1.

## 7. Conclusions

Experimental testing is the most appropriate way to verify the load-carrying capacity and stiffness of timber structures. Therefore, tests on bolted connections in squared and round timber with inserted steel plates, with different distances between the fastener and the loaded end, were performed.

Connections with inserted steel plates are also widely used in exterior timber structures (e.g., lookout towers [1], bridges [2], etc.) that are exposed to significant temperature and moisture content changes. Therefore, the effect of increased moisture content in timber on the load-carrying capacity of the connections was also analyzed in the experiment. 

The following partial conclusions can be drawn:

- Connection failure always occurred by timber splitting under the bolt, when the tensile strength perpendicular to the grain was exceeded.
- The average load-carrying capacity was lowest for the shortest fastener distance for both the squared timber and round timber. For specimens with higher distances, the average load-carrying capacity was higher.
- Considerable variability in the slip modulus can be seen in the evaluation. The slip modulus is higher for the round timber than for the squared timber, which is probably due to the higher bulk density of round timber.
- The decrease in real load-carrying capacity with increasing moisture content was also confirmed experimentally. The measured data could be approximated by a straight line using regression analysis.

The limitations of this research include the use of only one fastener in a row (bolt M12 8.8) and only one species of timber (spruce, C24 strength class), the limited number of test specimens (which affects the statistical evaluation) and the monotonic static testing.

Exterior structures are also predominantly loaded by wind, which has the characteristics of an alternating dynamic load [46]. On the basis of these facts, future research on this issue should incorporate experiments with cyclic and dynamic tests [8,47].

## Figures and Tables

**Table 1:** Dimensions of all specimen types for tension tests parallel to the grain.

Type of Specimen	H1	H2	H3	K1	K2	K3
Number of specimens [-]	15	13	13	5	5	5
Length of timber specimen [mm]	400	480	560	400	480	560
Height of timber specimen [mm]	2 × 60			2 × 60		
Width/diameter of timber specimen [mm]	120			⌀120		
Loaded end distance [mm]	140	180	220	140	180	220
Length of steel plate [mm]	320	360	400	320	360	400
Thickness of steel plate [mm]	10					
Width of steel plate [mm]	80					
Diameter of fastener [mm]	20					

**Table 2:** Values of bulk density, moisture content, maximum force, characteristic force and slip moduli for all types of specimens.

Comparison	H1	H2	H3	K1	K2	K3
ρ (AVG) [kg/m^3^]	417	411	414	458	455	431
ρ (SD) [kg/m^3^]	13.4	20.0	20.6	34.7	45.1	34.3
ρ (COV) [%]	3.2	4.9	5.0	7.8	9.9	8.1
w (AVG) [%]	12.8	12.6	13.0	13.2	12.8	12.8
w (SD) [%]	0.2	0.6	0.8	0.6	0.7	0.4
*F*_max,test_ (AVG) [kN]	66.10	75.43	72.63	69.98	85.81	81.62
*F*_max,test_ (SD) [kN]	3.94	8.26	4.84	8.04	10.79	5.28
*F*_max,test_ (COV) [%]	6.0	11.0	6.7	11.5	12.5	6.5
*F*_k,test_ [kN]	56.41	55.10	60.72	50.20	59.39	68.65
*F*_k,EC5_ [kN]	46.53					
*k*_i_ (AVG) [kN/mm]	5852	5931	5530	5889	5804	5519
*k*_s_ (AVG) [kN/mm]	6817	5644	6446	11,298	11,342	11,129

**Table 3:** Calculation of characteristic load-carrying capacity according to Eurocode 5.

Calculation according to Eurocode 5		
Fastener diameter [mm]		20
Characteristic fastener tensile strength [N/mm^2^]		800
Characteristic fastener yield moment [N/mm^2^]		579,281
Thickness of the timber side member [mm]		60
Characteristic timber density [kg/m^3^]		350
Characteristic embedment strength in timber [N/mm^2^]		22.96
Characteristic load-carrying capacity in single shear [N]	failure mode *a*	27,552
	failure mode *b*	23,264
	failure mode *c*	37,512
